# Redosing of Fluorescein Sodium Improves Image Interpretation During Intraoperative *Ex Vivo* Confocal Laser Endomicroscopy of Brain Tumors

**DOI:** 10.3389/fonc.2021.668661

**Published:** 2021-09-30

**Authors:** Irakliy Abramov, Alexander B. Dru, Evgenii Belykh, Marian T. Park, Liudmila Bardonova, Mark C. Preul

**Affiliations:** ^1^ The Loyal and Edith Davis Neurosurgical Research Laboratory, Department of Neurosurgery, Barrow Neurological Institute, St. Joseph’s Hospital and Medical Center, Phoenix, AZ, United States; ^2^ Department of Neurosurgery, Rutgers New Jersey Medical School, Newark, NJ, United States

**Keywords:** brain tumors, confocal laser endomicroscopy, fluorescein sodium, fluorescence-guided neurosurgery, glioblastoma, image interpretation, redosing

## Abstract

**Background:**

Fluorescein sodium (FNa) is a fluorescence agent used with a wide-field operating microscope for intraoperative guidance and with confocal laser endomicroscopy (CLE) to evaluate brain tissue. Susceptibility of FNa to degradation over time may affect CLE image quality during prolonged surgeries. This study describes improved characteristics of CLE images after intraoperative redosing with FNa.

**Methods:**

A retrospective analysis was performed using CLE images obtained *ex vivo* from samples obtained during tumor resections with FNa-based fluorescence guidance with a wide-field operating microscope. The comparison groups included CLE images acquired after FNa redosing (redose imaging group), images from the same patients acquired after the initial FNa dose (initial-dose imaging group), and images from patients in whom redosing was not used (single-dose imaging group). A detailed assessment of image quality and interpretation regarding different FNa dosage and timing of imaging after FNa administration was conducted for all comparison groups.

**Results:**

The brightest and most contrasting images were observed in the redose group compared to the initial-dose and single-dose groups (P<0.001). The decay of FNa signal negatively correlated with brightness (rho = -0.52, P<0.001) and contrast (rho = -0.57, P<0.001). Different doses of FNa did not significantly affect the brightness (P=0.15) or contrast (P=0.09) in CLE images. As the mean timing of imaging increased, the percentage of accurately diagnosed images decreased (P=0.03).

**Conclusions:**

The decay of the FNa signal is directly associated with image brightness and contrast. The qualitative interpretation scores of images were highest for the FNa redose imaging group. Redosing with FNa to improve the utility of CLE imaging should be considered a safe and beneficial strategy during prolonged surgeries.

## 1 Introduction

Intraoperative confocal laser endomicroscopy (CLE) is an emerging technology that evaluates brain tissue and pathologic tumor tissue *via* optical fluorescence-based interrogation. CLE technology produces images with resolution at cellular dimensions and displays them to the surgeon and pathologist in real time. CLE has the potential to maximize safe brain tumor resection and improve the positive yield and result time of tissue biopsy, particularly compared to frozen section biopsy, which may require significant time to return information vital to the progress of the surgical procedure. Unlike conventional clinical tools used for diagnostic work-up or in the operating room, CLE can provide real-time histopathologic information to distinguish tumor, non-tumor, and normal tissues *in vivo*. Thus, CLE can potentially improve tumor resection at the margin (1, 2).

To induce sufficient image contrast, either intravenous or topical exogenous fluorescence agents are used with CLE. Fluorescein sodium (FNa) was the first agent used for neurosurgical imaging based on its the previous success in gastroenterological CLE imaging (3). Since then, has been adopted as a fluorescence contrast for the first clinical-grade CLE system that has achieved US Food and Drug Administration (FDA) approval for use in neurosurgery. FNa has peak excitation at 494 nm and peak emission at 521 nm (4). When subjected to light at a wavelength of 560 nm, intravenously administered FNa emits a yellow-green fluorescence that marks the regions of blood-brain barrier (BBB) disruption (5, 6).

The initial clinical studies on research-grade CLE systems for brain tumor imaging with FNa were performed in the early 2010s in several centers (7–9). These studies involved the FNa administration protocol used in gastroenterology, specifically, the intravenous injection of an FNa bolus (5 ml) during the resection about 5 minutes before CLE imaging. During this same period, FNa was also being studied for use in neurosurgery as an intraoperative fluorescence contrast agent for wide-field guidance with an improved fluorescence filter set in the operating microscope (10, 11). These studies identified timing for FNa administration at the induction of anesthesia with a dose of 2 to 5 mg/kg. This timing and dosage have been used in a number of studies and have since become widely used in Europe and the United States.

We began assessing a CLE system for intraoperative neurosurgical use in 2016. The operation of this CLE system was based on the FNa Yellow 560 (Carl Zeiss Meditec AG, Oberkochen, Germany) fluorescence guidance protocol with FNa administration at the induction of anesthesia, a protocol that had already been established. The initial step in the use of the clinical-grade CLE system was to assess its performance in an *ex vivo* imaging environment. Our observations revealed that FNa signal intensity on CLE images was not always adequate to delineate the tissue at the time of surgical actions on the tumor when administered after anesthesia induction. This situation caused us to administer a second dose of FNa in order to achieve interpretable images and assess our imaging findings in greater detail. All fluorescence agents, including FNa, have a limited window of time during which their signal is optimal for visualization, after which they are subject to washout, metabolism, excretion, and photobleaching (12). Although some level of degradation is desirable so that fluorophores are excreted, a short half-life can compromise the contrast needed to accurately visualize tumor tissue morphology when longer observation is necessary or especially when actionable images are required for surgical decision-making. Therefore, effective deployment of CLE requires a refinement of dosing strategies and an appropriate choice of fluorescence dyes.

Preclinical animal-model studies and human clinical studies of brain tumors have reported promising results using FNa-based CLE for examining the histoarchitecture of and around brain tumors (13–18). However, FNa has a short half-life and is susceptible to degradation over time, which may affect CLE image interpretation during prolonged surgeries (19, 20). This shortcoming was circumvented with redosing with FNa, which we observed was beneficial for improving the clarity of CLE tissue visualization (18). Hence, redosing may provide histologic imaging information leading to more optimal tumor resection.

In this study, we describe these improved characteristics of CLE images when visualizing tumor tissue after FNa redosing. Through detailed image analysis, we assessed and quantified the enhanced quality of CLE images after FNa redosing in patients who underwent routine fluorescence-guided brain tumor resection using the Yellow 560 filter of the operating microscope together with *ex vivo* CLE imaging. This cohort was previously generally described by Belykh et al. (18). In this report, we analyze the differences in dosing strategies, diagnostic interpretation, and fluorescence signal strength and decay in addition to image quality. This report is the first to focus on FNa redosing to improve clinical CLE imaging of human brain tumor tissue.

## 2 Material and Methods

### 2.1 Study Design

Between August 2018 and May 2019, 47 adult patients (≥18 years) with brain tumors underwent FNa-guided surgery accompanied by *ex vivo* CLE imaging at Barrow Neurological Institute (Phoenix, Arizona) (18). The study was approved by the St. Joseph’s Hospital and Medical Center Institutional Review Board for Human Research (No. 10BN130), and informed consent was obtained from patients. FNa was administered intravenously at the induction of anesthesia at a dose of 2 mg/kg or 5 mg/kg and could be redosed as such up to four times. Multiple tissue samples obtained during tumor resection were imaged using a CLE station (CONVIVO, Carl Zeiss Meditec, AG, Oberkochen, Germany) in the same operating room. FNa redosing (5 mg/kg) was performed when image brightness during tumor resection was considered inadequate by the neurosurgeon, based on interpretation of the CLE images. CLE images were compared to the co-located conventional histologic hematoxylin and eosin (H&E)–stained images from the tissue biopsy preparations.

Seven patients had FNa redosed at some time during their surgery, but 1 case of redosing was excluded because the bandpass and longpass filters of the CLE system were used to manually increase the brightness of CLE images during surgery. All images from the remaining 6 redosing cases (4 gliomas, 1 metastasis, 1 choroid plexus carcinoma) were evaluated in this study. Each case was composed of multiple CLE optical biopsies or “spots” with a total of thousands of still CLE images.

Three FNa dose comparison imaging groups were generated for this analysis:

1) The redose imaging group (n=6) was composed of the cases where redose of FNa occurred during surgery. The redose occurred once, with FNa administered (2 to 5 mg/kg) according to the neurosurgeon’s assessment that the FNa signal did not sufficiently yield clear, interpretable, actionable CLE images.
2) The initial-dose imaging group (n=6) was composed of the same 6 cases as above, but the CLE images evaluated and compared were those obtained before the patient was redosed. Thus, the FNa signal was assessed in the same group of patients at their initial dosing at the beginning of the surgical procedure and then when the neurosurgeon deemed it necessary to acquire informative guidance to progress with tumor resection or assessment of the tumor resection area.
3) A single-dose imaging group (n=9) was composed of the 9 remaining cases of glioma where an FNa redose was not administered, but that were regarded to have image qualities similar to the initial-dose imaging group and where the neurosurgeon did not request FNa redose. The glioma cases were chosen because they were most similar in pathological character to the redose imaging group of cases.

### 2.2 Image Analysis

Our analysis included intensity value assessments of images, image quality at different FNa dosages, diagnostic accuracy, and the timing of imaging after FNa administration for all three groups.

The collected images were processed using Fiji open-source software (21). The specific intensity measurements were selected to evaluate image quality objectively. Each image was assessed according to two intensity modalities, brightness and contrast, reported in software program-defined optical density units. Brightness was defined as the mean gray value within the selected image. The value was calculated from the sum of the gray values of all the pixels in the selection divided by the number of pixels. Contrast was defined as the standard deviation (SD) of the gray values used to generate the mean gray value. Values of intensity modalities from different groups were compared to each other and for different FNa doses. Also, the mean brightness and contrast values of different biopsy spots were assessed by different imaging acquisition times after FNa administration.

### 2.3 Image Evaluation

To assess how the quality of a CLE image affects its interpretation, 7 reviewers with different levels of experience in interpreting CLE images assessed the images (2 experienced neurosurgeons, 1 experienced neuropathologist, 2 inexperienced neurosurgeons, 2 inexperienced non-neurosurgeons). Each reviewer reviewed and subjectively graded the CLE images for quality on a scale of 1 to 5, with 5 being the highest quality, optimally clear image. No information was provided to reviewers regarding diagnosis, frozen section description, preoperative imaging, surgical procedure, or lesion location. CLE images that were recognized to show abnormal cells or pathologic vessels were graded as “lesional”. “Normal” CLE images were defined by the presence of overwhelming or total normal brain tissue within the image. CLE images were considered “noninterpretable” based on an inability or difficulty to distinguish morphological structures. Images showing obvious artifacts, such as probe motion, acquisition problems, etc., were excluded. The overall interpretation of CLE images and H&E histologic sections was compared among different groups to define the diagnostic accuracy.

### 2.4 Statistical Analysis

Statistical analysis was performed using GraphPad Prism 9 (GraphPad Software, Inc., La Jolla, CA). Continuous variables were presented as means with standard deviations (SDs). Categorical variables were described using counts and percentages. The differences in intensity values and timing of imaging between the two groups were assessed with a Mann-Whitney U-test. Categorical variables in subgroups were compared with the chi-square test. The Spearman correlation coefficient was used to assess the association between the timing of imaging and intensity values. A P value < 0.05 was considered significant.

## 3 Results

### 3.1 Descriptive Analysis

From 8854 available images, 1503 were excluded due to blackout signal or motion artifacts. Additionally, 335 images acquired with the CLE Z-stack image acquisition mode were excluded because the automatically recorded range of images from different focal depths represents an alternate image acquisition process that obstructs the analysis of image intensity. A total of 7016 images from 49 CLE imaging biopsies from the three imaging groups were identified for analysis: 1) the redose imaging group had 12 biopsies (2184 images); 2) the initial-dose imaging group had 18 biopsies (2535 images); and, 3) the single-dose imaging group had 19 biopsies (2297 images).

FNa dosing was 5 mg/kg per the redosing protocol for all 12 CLE imaging biopsies from the redose imaging group. FNa was administered at a dose of 5 mg/kg in 8 of 18 biopsies (44%) in the initial-dose imaging group and 2 of 19 biopsies (12%) in the single-dose imaging group, whereas 10 of 18 CLE imaging biopsies (56%) in the initial-dose imaging group and 17 of 19 CLE imaging biopsies (88%) in the single-dose imaging group were acquired at a FNa dose of 2 mg/kg.

CLE optical biopsy acquisition occurred at a mean (SD) of 6.4 (3.8) min after FNa administration in the redose imaging group, at 93.9 (50.1) min in the initial-dose imaging group, and at 123.2 (35.9) min in the single-dose imaging group.

### 3.2 Analysis of Image Intensity

#### 3.2.1 Overall

**Figure 1:**
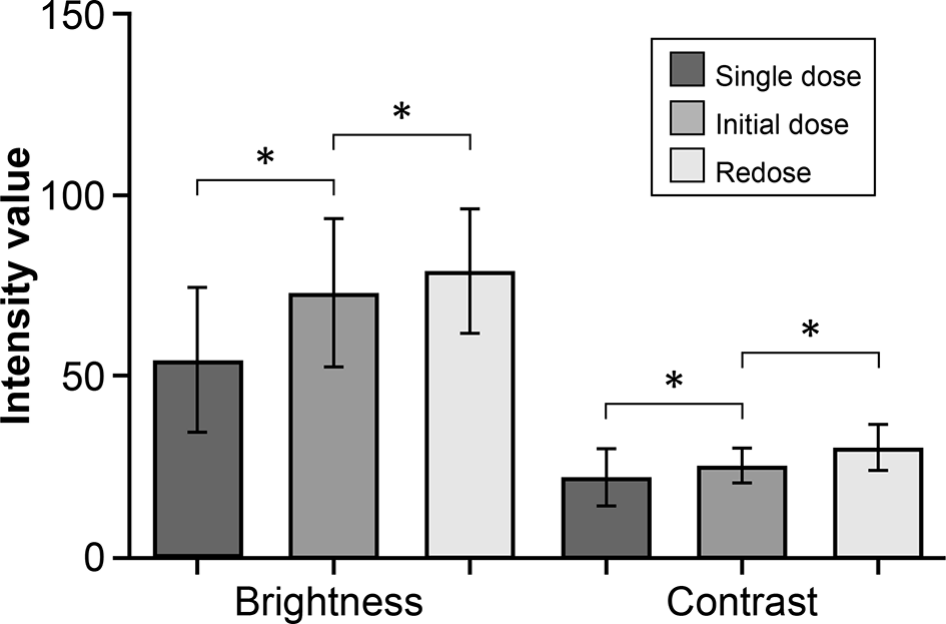
Bar plot showing a comparison of the mean (SD) intensity modalities in the fluorescein sodium redose, initial-dose, and single-dose groups. The top and bottom whisker marks indicate the SD. *P < 0.001. The mean (SD) values of brightness and contrast of images are 79.1 (17.2) and 30.5 (6.3), respectively, in the redose group; 73.1 (20.4) and 25.4 (4.9) in the initial-dose group; and 54.6 (20.0) and 22.2 (7.9) in the single-dose group. The values are reported in units of optical density defined by Fiji open-source analytic software. Brightness is the mean gray value, calculated by the sum of the gray values of all the pixels in the selection divided by the number of pixels. Contrast is the SD of gray values used to generate the mean. SD, standard deviation. *Used with permission from Barrow Neurological Institute, Phoenix, Arizona*.

**Figure 2:**
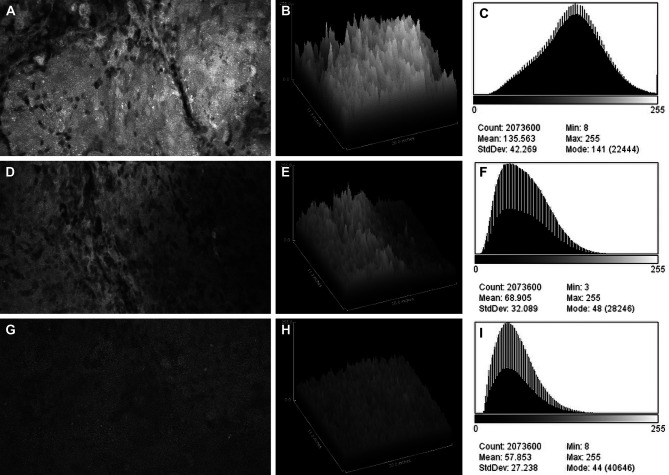
Confocal laser endomicroscopy (CLE) images and data from **(A–C)** the fluorescein sodium redose, **(D–F)** initial-dose, and **(G–I)** single-dose groups. A CLE image **(A, D, G)** from each group is shown with a corresponding three-dimensional surface plot of pixel intensities **(B, E, H)** and a histogram of intensity values **(C, F, I)**. Count, number of pixels; Mean, brightness; StdDev, contrast; Min and Max, minimum and maximum gray values within the image; Mode, most frequently occurring gray value within the selection corresponds to the highest peak in the histogram. *Used with permission from Barrow Neurological Institute, Phoenix, Arizona*.

The brightness and contrast values for images in the redose imaging group were higher than for images in the initial-dose imaging group (P<0.001) (**Figures 1**, **2**). Similarly, the images in the initial-dose imaging group had higher values of intensity modalities than in the single-dose imaging group (P<0.001).

#### 3.2.2 FNa Dosing

**Figure 3:**
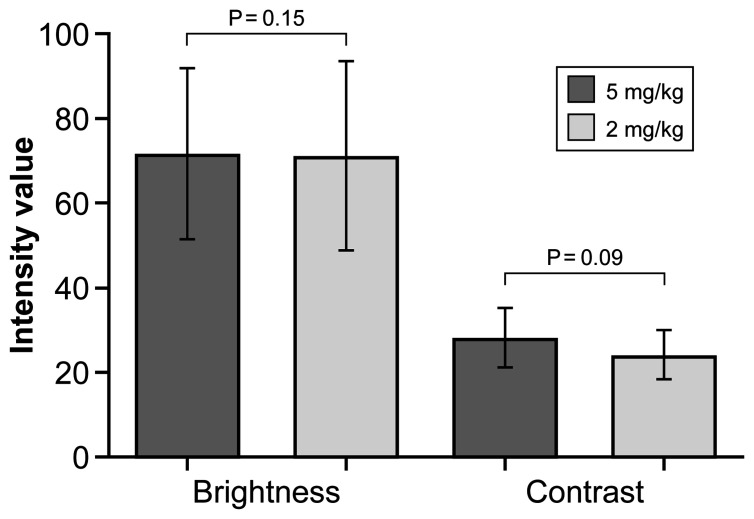
Bar plot showing mean (SD) intensity modalities for confocal laser endomicroscopy images acquired after a fluorescein sodium dose of 5 mg/kg or 2 mg/kg. Whisker bars indicate the SD. The values are reported in units of optical density defined by Fiji open-source analytic software. Brightness is the mean gray value, calculated by the sum of the gray values of all the pixels in the selection divided by the number of pixels, and contrast is the SD of gray values used to generate the mean. *Used with permission from Barrow Neurological Institute, Phoenix, Arizona*.

Overall, mean (SD) brightness and contrast of images acquired at a FNa dose of 5 mg/kg were 71.7 (20.1) and 28.3 (7.0), respectively; at a dose of 2 mg/kg, results were 71.3 (22.4) and 24.3 (5.8), respectively. These different doses of FNa did not significantly affect the brightness (P=0.15) or contrast (P=0.09) of the CLE images (**Figure 3**). Images from the initial-dose imaging group were brighter and had better contrast than in the single-dose imaging group regardless of the difference of FNa dose (P<0.001). Further analysis on the timing of imaging after administration of FNa in these groups revealed a significant difference: images from the initial-dose imaging group were acquired at a shorter mean (SD) time when compared to the single-dose imaging group (93.9 [50.1] min *vs.* 123.2 [35.9] min, respectively; P=0.002).

#### 3.2.3 Timing of Imaging

**Figure 4:**
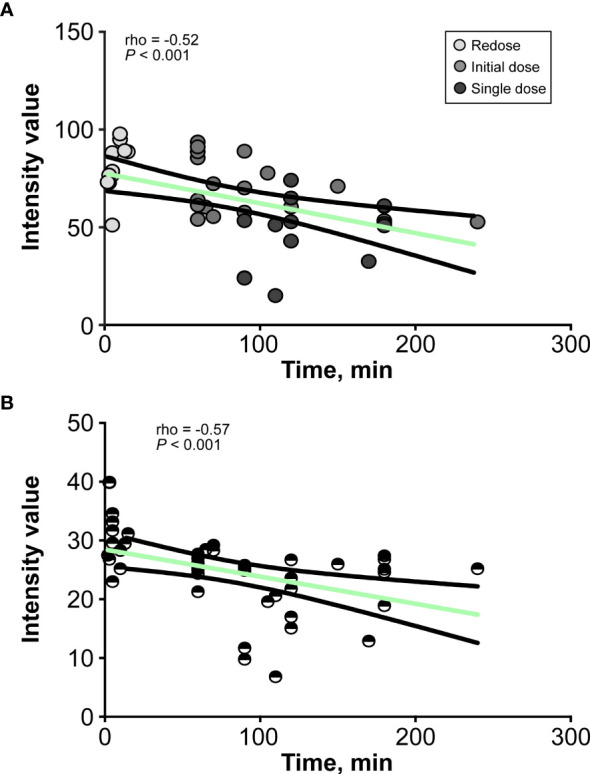
Line graphs show the relationship between **(A)** the brightness and **(B)** the contrast of the confocal laser endomicroscopy image and the decay of fluorescein sodium in minutes. The values are reported in units of optical density defined by Fiji open-source analytic software. Brightness is the mean gray value, calculated by the sum of the gray values of all the pixels in the selection divided by the number of pixels, and contrast is the standard deviation of gray values used to generate the mean. *Used with permission from Barrow Neurological Institute, Phoenix, Arizona*.

The brightness and contrast values for each CLE optical biopsy from the three groups and the timing of imaging are shown in **Table 1**. The analysis of intensity values at different imaging time points after FNa administration revealed a moderate correlation between the imaging timing and image brightness for CLE biopsies (rho = -0.52, P<0.001) (**Figure 4A**). Similarly, a moderate correlation was observed between the imaging timing and image contrast for CLE biopsies (rho = -0.57, P<0.001) (**Figure 4B**).

**Table 1:** Image brightness, contrast, and timing for each CLE optical biopsy by FNa dosing groups.

FNa Group:CLE biopsy no.	Brightness*	Contrast*	Time after FNa dose, min
Redose			
1	88.2	33.2	5
2	95.0	25.2	10
3	88.7	31.2	15
4	72.9	26.8	3
5	76.8	34.6	5
6	51.2	31.7	5
7	51.1	23.0	5
8	78.7	29.6	5
9	76.7	39.9	5
10	73.2	27.5	3
11	97.8	28.3	2
12	89.2	29.5	10
Initial dose			
1	52.8	25.2	13
2	53.4	25.1	180
3	71.0	26.0	150
4	64.0	24.4	60
5	60.4	28.5	65
6	55.5	29.2	70
7	54.1	21.3	60
8	61.3	26.2	60
9	72.3	28.3	70
10	57.8	25.8	90
11	60.4	26.7	120
12	85.6	27.0	60
13	88.7	25.6	60
14	93.7	25.1	60
15	91.2	27.7	60
16	89.0	25.3	90
17	70.2	24.9	90
18	77.8	19.6	105
Single dose			
1	60	27.4	180
2	52	24.6	180
3	61	26.8	180
4	43	15.1	120
5	65	21.8	120
6	52.9	17	120
7	74.1	23.6	120
8	24.2	9.8	90
9	24	11.7	90
10	15.1	6.8	110
11	32.6	12.9	170
12	53.4	25.7	90
13	51.3	20.6	110
14	50.8	18.9	180
15	63.3	26.2	90
16	67.8	24.7	90
17	68.8	25.5	90
18	71	27	90
19	66	25	120

*Values are reported in units of optical density defined by Fiji open-source analytic software. Brightness is the mean gray value, calculated by the sum of the gray values of all the pixels in the selection divided by the number of pixels, and contrast is the standard deviation of gray values used to generate the mean.

### 3.3 Interpretation of Images

A qualitative assessment of images on a 1-to-5 scale showed that the highest mean (SD) scores were associated with images from the redose imaging group (4.5 [0.6]), followed by the initial-dose imaging group (2.3 [0.2]) and single-dose imaging group (2.3 [0.1]). Results of interpretation of CLE biopsies for each reviewer are shown in **Table 2**. When using images from the redose imaging group, the diagnostic accuracy was 83% regardless of reviewer experience. As the mean timing of imaging increased, the percentage of accurately diagnosed images decreased among reviewers and was significantly different for both experienced neurosurgeons (both P=0.03) and an experienced neuropathologist (P=0.03). No significant difference was observed in the diagnostic accuracy of groups by either inexperienced neurosurgeon (P=0.2 and P=0.1) or by the inexperienced non-neurosurgeons (both P=0.3).

**Table 2:** Diagnostic accuracy of interpretation of CLE biopsies by reviewer.

	FNa Dosing Group			P value
	Redose, n=12	Initial dose, n=18	Single dose, n=19	
Mean timing, min*	6.4 ± 3.8	93.9 ± 50.1	123.2 ± 35.9	
Diagnostic accuracy, no. (%)				
Experienced neuropathologist	10 (83)	13 (72)	9 (47)	0.03
Experienced neurosurgeon	10 (83)	13 (72)	9 (47)	0.03
Experienced neurosurgeon	10 (83)	13 (72)	9 (47)	0.03
Inexperienced neurosurgeon	10 (83)	7 (39)	10 (53)	0.2
Inexperienced neurosurgeon	10 (83)	14 (78)	11 (58)	0.1
Inexperienced non-neurosurgeon	10 (83)	10 (56)	12 (63)	0.3
Inexperienced non-neurosurgeon	10 (83)	11 (61)	12 (63)	0.3

CLE, confocal laser endomicroscopy; FNa, fluorescein sodium.

*Mean timing of CLE biopsy imaging after FNa administration.

## 4 Discussion

FNa, which has been approved by the FDA for use in neurosurgery, is most frequently used in cerebrovascular and neuro-oncological procedures and is now also used with CLE. Although initially limited to ophthalmology, the use of FNa in neurosurgical applications was first reported in 1948 for localizing brain tumors (22). Its use in neurosurgery has expanded since the introduction of integrated fluorescence filters, such as the Yellow 560 filter, in clinical-grade operating microscopes (6, 10, 11, 19). Intravenous administration of FNa results in the extravasation of the compound in areas of disrupted BBB, such as those caused by a tumor (17).

CLE imaging technology allows neoplasm assessment through the presence of augmented fluorescence signals and real-time identification of abnormal tissue (15, 18). These novel handheld portable imaging systems have cellular-dimension resolution and can even allow visualization of subcellular structures. Optimization of these systems is being explored for maximal or better-informed tumor resection or tissue interrogation, particularly at the tumor margin, while preserving adjacent healthy brain tissue. Such technology may inform whether tumor invasion has occurred into exquisitely functional cortex, and thus whether resection should be halted.

Previously, our prospective clinical study examined the diagnostic accuracy of *ex vivo* intraoperative FNa-based CLE optical biopsies (18). In this study, we analyzed and described the improved features of the CLE images after FNa redosing and assessed image quality as the decay of FNa increased. Currently, FNa redosing is allowed at our institution under the parameters of institutional review board protocol for neurosurgical procedures, whereas in Europe, FNa redosing is not specified under the regulations and is therefore not performed.

Our study has shown improved CLE imaging results after the redosing of FNa during surgery. The first FNa dose was administered according to the wide-field fluorescence guidance protocol employing the operating microscope Yellow 560 module at the start of the anesthesia. CLE imaging could be performed successfully at this time parallel to the Yellow 560 imaging; however, in cases of insufficient FNa signal intensity and poor CLE image quality, redosing FNa (similar to its administration as per initial gastrointestinal protocol) can be performed to improve CLE image brightness, contrast, and overall quality for interpretation. Occasionally, CLE imaging acquisition occurs in a delayed fashion following FNa administration, which may result in decreased image quality, (i.e., darker, less interpretable images) as assessed by the operating neurosurgeon. In such cases, we have redosed the patient with FNa. We have not redosed patients with FNa multiple times, but our surgical protocol would allow up to four redosings at the low-dose parameters prescribed.

### 4.1 Image Intensity Analysis

The pharmacokinetics of intravenous FNa redistribution is nearly instantaneous, compared to the oral pathway and the time required for 5-aminolevulinic acid (5-ALA) distribution (23), which makes redosing of the latter infeasible. The 5-ALA metabolite induces protoporphyrin IX (PpIX) accumulation in tumor cells and takes longer to produce fluorescence than FNa (24). Intensity analysis is measured differently for 5-ALA than FNa because 5-ALA fluorescence emanates from cells, whereas FNa accumulates in the extracellular space. After oral administration of 5-ALA (20 mg/kg), peak fluorescence is expected in about 6 to 8 hours (25–27), with a fluorescence accumulation satisfactory for operation at around 3 hours. Fluorescence is eliminated with a terminal half-life of approximately 1 to 3 hours (27).

This study assesses a central question relevant to the use of CLE for neurosurgery, which is a focus of the few centers currently permitted to use this technology. Only recently has one system been approved by the US FDA and European Medicines Agency for use in clinical neurosurgical work, which our institute and several other European centers are using. However, only in the United States is redosing of FNa permitted, thus our group of patients provides valuable data to guide administration of FNa. Ideally, the initial dose should be closer to the time of imaging as we have observed a time-associated decay of FNa fluorescence, with a corresponding decrease in CLE image quality. As a result, we investigated the benefit of FNa redosing because of its time-dependent degradation.

In the European centers using FNa, and initially in our center, FNa was administered shortly after the induction of anesthesia. However, after many minutes had passed in the performance of the craniotomy and start of tumor resection, it was observed that the CLE imaging was not optimal and could not be interpreted reliably. The FNa administration technique was then changed at our center so that FNa was administered nearer to the time that tumor resection was begun. Even after this change in administering FNa, many minutes may pass from the start of the tumor resection to the final stages of resection in which tumor extensions are evaluated in restricted or remote locations or where the tumor margins are unclear or suspicious within the resection bed. These final circumstances in tumor surgery, especially for invasive gliomas, often involve critical surgical decisions of whether to extend or curtail the surgical maneuvers toward optimizing the resection. During this time, the signal of FNa may deteriorate significantly and may require the administration of a redose to produce actionable CLE images. Redosing appears to be safe and effective, because the initial FNa dose is relatively low, and the resulting redose of FNa may provide exquisitely improved histological imaging information.

**Figure 5:**
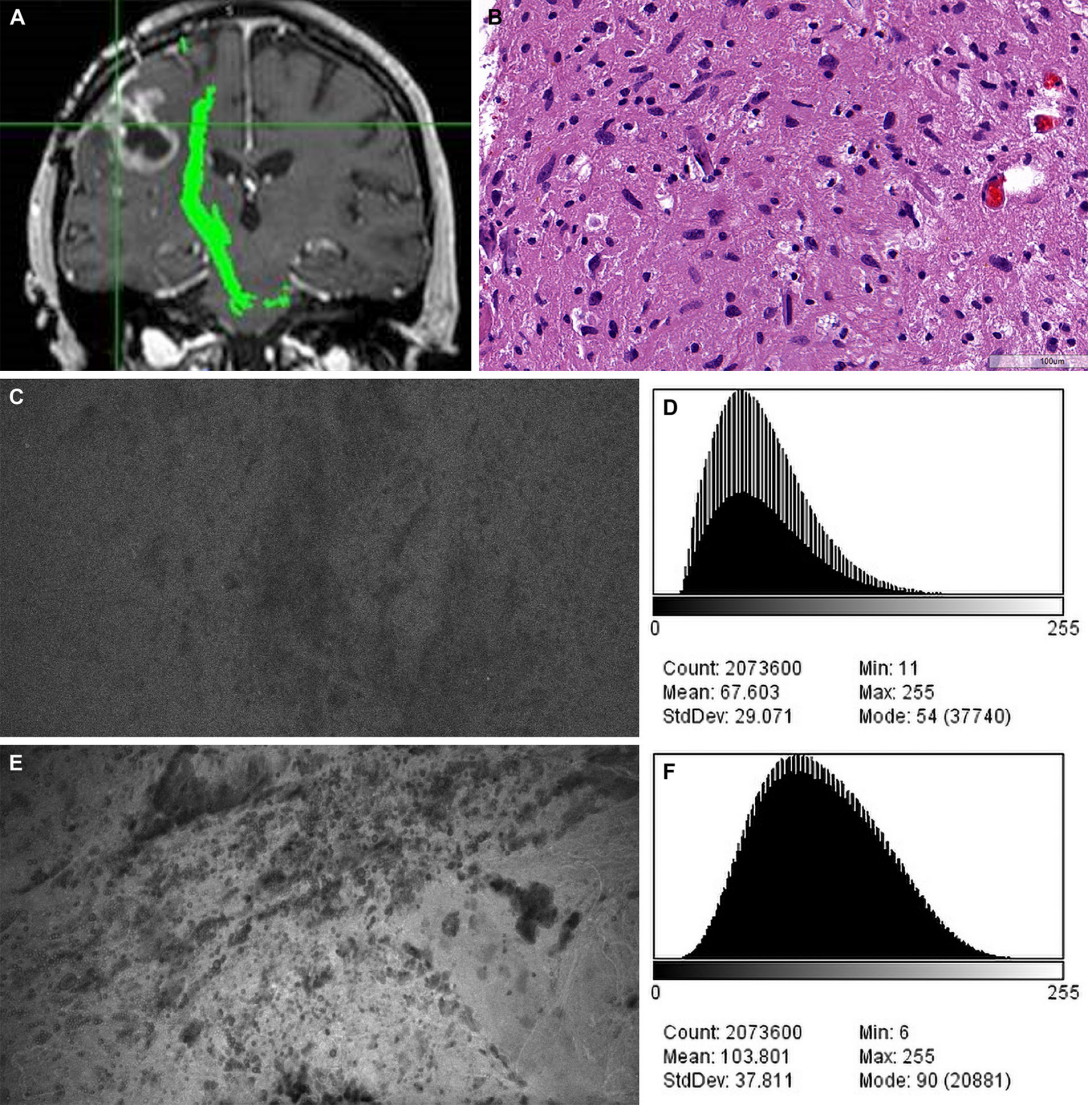
Case example of confocal laser endomicroscopy (CLE) images acquired after fluorescein sodium (FNa) redosing. **(A)** Coronal T1-weighted magnetic resonance image with contrast. Biopsy specimens were obtained from the enhancing tumor in the left frontal lobe. **(B)** A diagnosis of high-grade glioma was made on the basis of H&E staining of the biopsy specimens. **(C)** A CLE image acquired 95 minutes after initial FNa injection lacks clarity and was regarded as noninterpretable. **(D)** A histogram corresponding to the image in C shows intensity values of the CLE image acquired after the initial FNa dose. **(E)** A marked increase in brightness and contrast of the CLE image was observed after FNa redosing. **(F)** A histogram corresponding to E shows the intensity values of the CLE image acquired after FNa redosing. Count, number of pixels; Mean, brightness; StdDev, contrast; Min and Max, minimum and maximum gray values within the image; Mode, most frequently occurring gray value within the selection corresponds to the highest peak in the histogram. *Used with permission from Barrow Neurological Institute, Phoenix, Arizona*.

**Figure 6:**
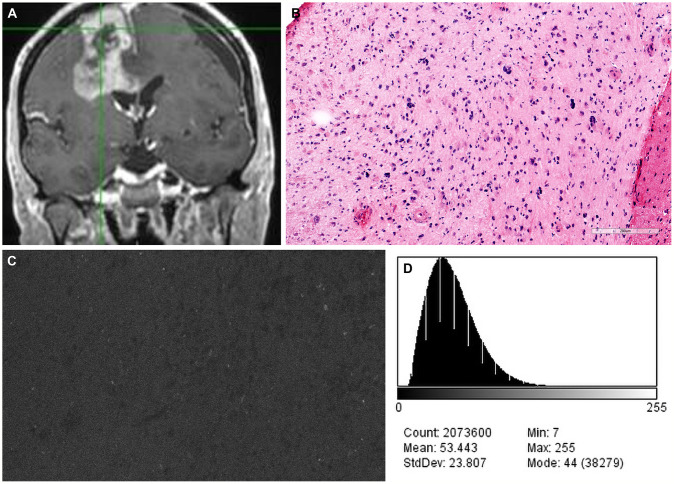
Case example of confocal laser endomicroscopy (CLE) images acquired after a single dose of fluorescein sodium (FNa) before the concept of redosing was introduced. **(A)** Coronal T1-weighted magnetic resonance image with contrast. Biopsy specimens were obtained from the enhancing tumor in the left frontal lobe. **(B)** A diagnosis of high-grade glioma was made on the basis of H&E staining of biopsy specimens. **(C)** A CLE image acquired 180 minutes after FNa injection is dark and noninterpretable. **(D)** A corresponding histogram shows the intensity values of the CLE image. Count, number of pixels; Mean, brightness; StdDev, contrast; Min and Max, minimum and maximum gray values within the image; Mode, most frequently occurring gray value within the selection corresponds to the highest peak in the histogram. *Used with permission from Barrow Neurological Institute, Phoenix, Arizona*.

Our results demonstrated that among the three comparison groups, the brightest and most contrasting images were observed in the redose imaging group, when compared to the initial-dose imaging group and single-dose imaging group (P<0.001) (**Figure 5**). However, a significant difference in intensity values was observed between images after the initial FNa dose and images from the selected cases where an FNa redose was not used (P<0.001). The single-dose imaging group was initially hypothesized to have similar image qualities to the initial-dose imaging group because the acquisition of CLE images occurred at a relatively similar time after administration of a single FNa dose in both groups (93.9 [50.1] min *vs.* 123.2 [35.9] min). However, although we tried to match these groups for imaging timing, the difference of 30 minutes between these groups was substantial enough to affect the quality of images (**Figure 6**). This observation may be supported by the pharmakokinetics of FNa. We made every effort to exactly match the groups, and 30 minutes difference was as close as possible for the groups. These variances are the result of timely decisions made at surgery by the operating surgeon viewing and assessing the CLE images.

### 4.2 Timing of CLE Imaging

Approximately 80% of FNa binds weakly to plasma proteins, mainly to albumin, and the remaining 20% is the free salt (28, 29). Therefore, both forms of the fluorophore are initially present in systemic circulation before permeating through a damaged BBB and concentrating at the tumor site (30). Within 1 hour of intravenous administration, FNa is rapidly metabolized in the liver to fluorescein monoglucuronide, which also exhibits fluorescent properties but to a much lesser degree (20, 28, 31). Blair et al. (20) have reported that 4 to 5 hours after intravenous FNa injection, almost all plasma fluorescence is due to the weakly illuminating fluorescein monoglucuronide. Plasma elimination half-lives of fluorescein and fluorescein monoglucuronide are 23.5 and 264 minutes, respectively (20), with complete renal clearance by 24 to 32 hours (28, 32).

Given the time-dependent kinetics and limited signal duration of FNa and 5-ALA, the fluorescence strength of a given fluorophore can degrade over the course of an operation. The analysis of all CLE images obtained at different time intervals showed a gradual decrease in image quality as the signal decayed, resulting in darker images (P<0.001) and worse contrast (P<0.001) (**Figure 4**). In other words, FNa redosing was beneficial for the maintenance of image quality and higher diagnostic accuracy. However, due to the nonselective passive permeability of FNa, it may induce false-positive fluorescence in areas with surgical damage and edema that are classically present at tumor margins (19).

The problem of image quality degradation over time may be also related to the *ex vivo* nature of the analysis; with *in vivo* acquisition, this problem might be attenuated. *Ex vivo* CLE analysis could involve additional time to prepare the tissue sample for imaging. However, our setup for CLE imaging was directly within the operating room with tissue samples imaged within seconds after removal. Thus, we believe that these data reflect closely what may occur with human *in vivo* FNa CLE imaging. We have observed similar results with repeated FNa dosing during *in vivo* imaging in animal tumor studies (17).

### 4.3 CLE Image Interpretation

Analysis of the reviewers’ interpretations of CLE images showed that the diagnostic accuracy for CLE biopsies obtained after redose was 83% regardless of the reviewer’s level of experience with CLE or in interpreting CLE images. This reveals obvious interpretable improvement in image quality. When analyzing images, the reviewers were blinded to patient and surgical data; thus, their CLE diagnostic accuracy would be expected to substantially increase if other clinical and surgical information were provided as normally occurs in patient management and operative situations. All 7 reviewers demonstrated a higher percentage of accurate diagnoses in the analysis of redose imaging group versus initial-dose imaging group and single-dose imaging group; however, there was a significant increase in diagnostic accuracy across the 3 groups for the experienced neurosurgeons and neuropathologist (**Table 2**). This finding likely indicates a more rigorous interpretation of CLE images by more experienced users familiar with the CLE system and images. Regardless, the longer the time from FNa dosing to CLE imaging generates poorer or less interpretable images. Similarly, subjective assessment of image quality showed that images from the redose imaging group were interpreted as the highest quality, which is supported by our intensity analysis. Standard deviation scores for image quality assessment were relatively low regardless of the reviewers’ experience, which indicates that the improvement in the quality of images was obvious.

### 4.4 Safety Profile of FNa

The safety profile of FNa is well established, as confirmed in cross-sectional surveys, randomized studies, and prospective cohorts (33–38). Of note, patients with severe liver diseases may experience prolonged yellow skin discoloration after FNa administration due to slower excretion of the fluorophore (22). Rare anaphylactic reactions from FNa injection have been reported (39), but it has been less of a concern in recent neurosurgical applications, especially because FNa can be administered at lower doses using specific filters like Yellow 560 (11). In addition, FNa is devoid of the drawbacks associated with the metabolic fluorophore 5-ALA, which are the high cost and the need for patients to avoid sunlight and bright indoor light for 24 hours after administration due to porphyrin-related skin sensitivity (40, 41).

**Figure 7:**
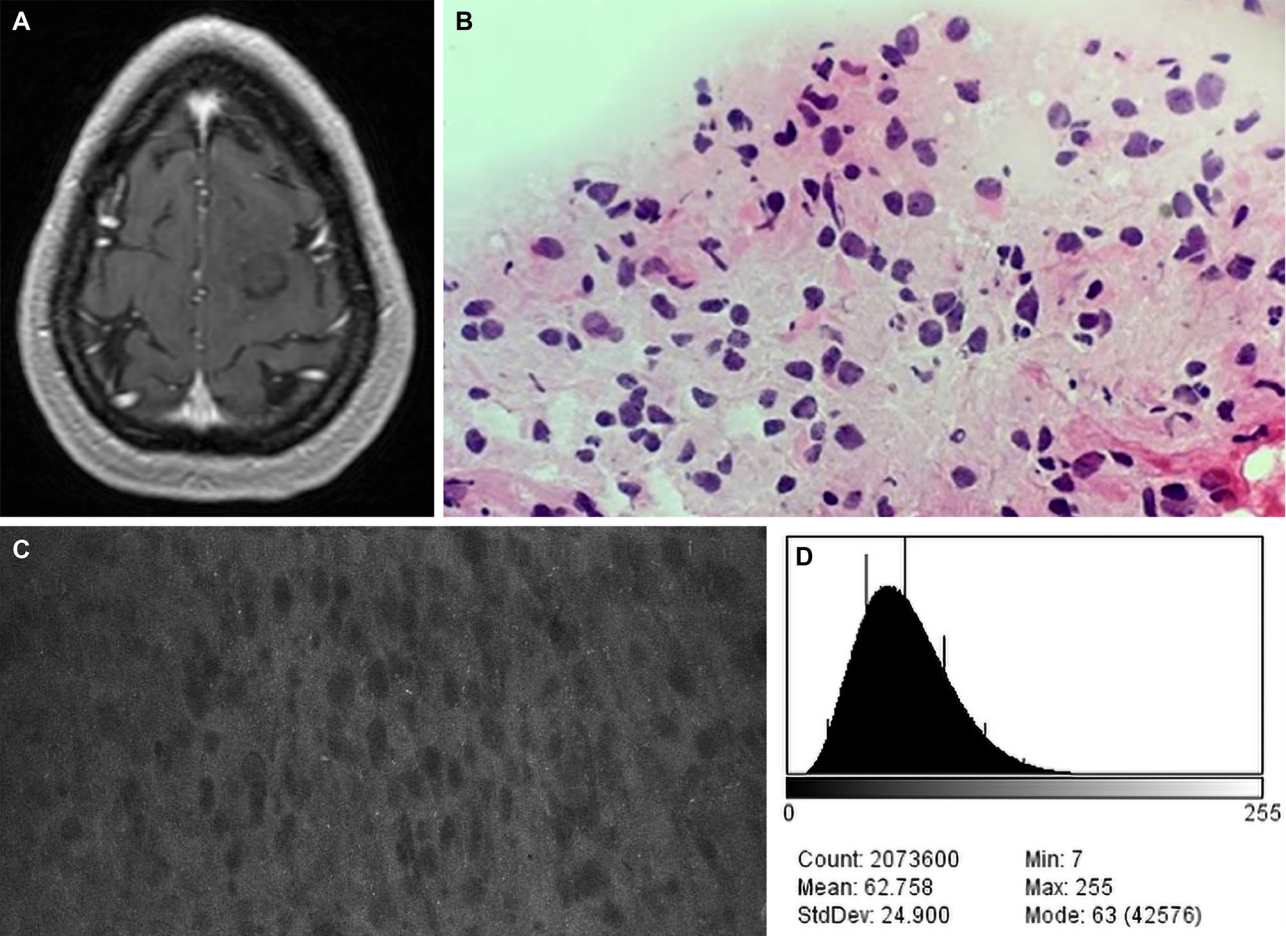
Case example of CLE images acquired at a fluorescein sodium (FNa) dose of 40 mg/kg. **(A)** Axial T1-weighted magnetic resonance image with contrast. Biopsy specimens were obtained from the nonenhancing tumor in the left frontal lobe. **(B)** A diagnosis of low-grade glioma was made on the basis of H&E staining of biopsy specimens. **(C)** A CLE image acquired 90 minutes after FNa injection appears to be relatively dark, but tumor cells are still present. **(D)** A corresponding histogram shows the intensity values of the CLE image. Count, number of pixels; Mean, brightness; StdDev, contrast; Min and Max, minimum and maximum gray values within the image; Mode, most frequently occurring gray value within the selection corresponds to the highest peak in the histogram. *Used with permission from Barrow Neurological Institute, Phoenix, Arizona*.

We reported that 1 patient who was administered 40 mg/kg of FNa experienced a yellow tinging of the skin that resolved within 48 hours, with no other adverse reactions (18, 42) (**Figure 7**). However, it is not clear whether FNa doses higher than administered in our study would result in a longer duration or a higher quality of signal since we did not observe significantly different brightness (P=0.15) and contrast (P=0.09) values between the different low FNa doses administered to the patients of this study.

### 4.5 Other Fluorophores

Recent studies have reported preliminary work on the simultaneous use of FNa and 5-ALA to provide fluorescent effects that would better discriminate pathologic tissue (27, 28). Suero Molina et al. (43) used a long-pass filter that allowed light to pass between 545 and 740 nm to allow simultaneous visualization of FNa and PpIX fluorescence with appropriate light illumination. The investigators reported that FNa provided an enhanced background for 5-ALA fluorescence; tumor tissue was detected as a red-orange fluorescence due to dual-labeling of PpIX and FNa (43). Whether simultaneous FNa and 5-ALA administration could provide additional benefit to help surgically manage invasive brain tumors merit further investigation, especially as it relates to CLE-based tissue interrogation.

Other intraoperative fluorophore options include indocyanine green (ICG) and topical fluorescence stains. ICG has prior applications in neurosurgery, most commonly in cerebrovascular neurosurgery for angiography (28, 44). However, unlike FNa and 5-ALA, ICG is a near-infrared (NIR) fluorophore (peak excitation = 805 nm, peak emission = 835 nm), which leads to enhanced tissue penetration and less absorption of light by hemoglobin and other tissue proteins (45). ICG binds to plasma proteins, thereby remaining intravascular; however, in cases of BBB breakdown, ICG was taken up by the tumor cells (46). In a technique known as second-window ICG with the enhanced permeability and retention effects of both intra- and extra-axial neoplasms, administration of ICG 16 to 30 hours before surgery as a bolus allowed enhanced visualization of many brain tumors (47).

Currently, although CLE systems are used in research environments, ICG and other NIR fluorophores cannot be detected within the laser operating range of the clinical-grade FDA-approved CLE imaging system used in this study. Additional limitations of these fluorophores include a high false-positive rate due to the unspecific labeling of any tissue with altered vasculature, the need for specific NIR sensors in microscopes and/or exoscopes, and washout from any ambient light source. Topical contrast agents like acriflavine, acridine orange, and cresyl violet have been used for CLE (48), but they may not be suitable for *in vivo* application because of possible mutagenic effects (49). Currently, only FNa is approved for use with CLE (15), but next-generation targeted fluorophores may be appropriate for redosing or re-administration intraoperatively on demand.

Simultaneous imaging of different fluorophores is possible, but they cannot necessarily be administered simultaneously, as 5-ALA must be administered orally hours before the surgery, and ICG is best administered hours before surgery for optimal uptake by tumor cells (46). Although 5-ALA and ICG appear to label tissue at the cellular level, there are no approved CLE systems for their use in neurosurgery. Therefore, considerations of redosing these fluorophores with a CLE system are irrelevant at this time, and for 5-ALA such consideration would seem to be especially complex. Thus, although FNa is not taken up by cells in the same way as 5-ALA or ICG, in our experience, CLE with FNa contrast labelling demonstrates reasonable and useful histoarchitectural feature recognition and a good correlation of pathology compared to H&E stained histologic examination. Further delineation of the optimal timing of administration of these fluorophores alone and together with FNa is warranted.

### 4.6 CLE Technique Incorporation Into Surgery

CLE-guided tumor surgery using FNa fluorescence contrast is an emerging technology. CLE is not wide-field imaging such as that obtained by an operating microscope with fluorescence detection modules. Wide-field fluorescence imaging with the operating microscope does not show cellular resolution, but reveals a greater detection area of fluorescence signal, which is often vague or indeterminate at the margins. The so-called “contrast labeling” of tissue seen in FNa-based CLE provides histologic imaging information on the cellular level. CLE provides cellular resolution but is limited to a small field of view of histoarchitecture. It is also more sensitive to changes in fluorescence signal intensity and quality. Because this imaging system is so new, there has not been time to validate CLE imaging compared with wide-field operating microscope FNa imaging. These studies, which would seem to be advantageous for combining the two imaging techniques, especially for examination at the margins of invasive tumors, are underway. Currently, there are no guidelines or protocols as to when the initial dose of FNa should be administered in CLE-guided tumor surgery, and no other studies on the “redosing” of FNa exist. The relatively short half-life of FNa may affect the quality of images obtained with CLE; therefore, it is important to identify the optimal timing for planning of resection and intraoperative visualization of tumor histoarchitecture.

Unlike wide-field FNa-guided tumor resection, FNa-based CLE-guided resection provides surgeons and pathologists with histopathological information in real time, allowing them to distinguish between tumor and normal brain tissue and increasing the potential for an optimal tumor resection. The small field of view of CLE may not allow an exact tissue diagnosis, but it can help identify abnormal histoarchitecture. CLE offers an “optical biopsy,” because of its ability to image at cellular or subcellular resolution, and it can therefore provide greater positive yield of tissue biopsies. CLE does not require tissue biopsy, as opposed to the tissue biopsy acquired with the use of the operating microscope and fluorescence detection modules. The surgeon and pathologist can interact in real time to interpret the CLE imaging produced on the fly.

Although the current FNa-based CLE system can be used without wide-field FNa fluorescence operating microscope technique, it would seem that the most reasonable and optimal use of FNa CLE would be its incorporation as an adjunctive tissue interrogation technology to surgery conducted with FNa *via* visualization using the operating microscope. It is also important to recognize that despite the benefit of FNa redosing seen in *ex vivo* conditions reported here, such redosing may interfere in the discrimination of the intra-axial tumoral tissue and normal brain tissue in the presence of surgical trauma during FNa-guided surgery. There may be iatrogenic leakage of FNa into the extracellular space, especially in the areas such as the tumor resection bed. In the first few seconds to minutes after administration, FNa defines the tumor well as viewed with the fluorescence module of the operating microscope, especially for high-grade gliomas, but then signal begins to emanate from surrounding surgical tissues, such as muscle or even scalp, as FNa distributes. Such FNa signal overage may become overwhelming when viewed through the operating microscope. Theoretically, CLE should be more sensitive in detecting changes in FNa signal, and thus the rationale to use repeated but low doses of FNa that will not overwhelm the fluorescence viewing system of the operating microscope. These redoses would be used when the CLE images have been determined to be inadequate for histoarchitecture discrimination and surgical decision-making.

### 4.7 Study Limitations

In the present sample of cases, only 6 patients were studied who underwent FNa reinjection, which is a low number for a powerful statistical analysis. However, although only 6 redosing cases were included, thousands of images were analyzed in each group. The cases studied mainly included gliomas and not tumors such as meningiomas, where there may be different FNa fluorescence characteristics. The principal use of CLE may find similar application in meningiomas where it may be used to image the dura to detect tumor invasion. FNa signal decay and retention is dependent on BBB permeability and hence may be different for various histopathological types of lesions. However, the CLE system was designed to be used mainly with primary invasive brain tumors.

Redosing of FNa was performed relatively late in our study period, as the idea had not been contemplated early on. At other times, redosing was not done because there was limited tissue sampling or no further tissue sampling was performed. At other times, the surgeon did not want to redose. Planning a future prospective multicenter study is reasonable, with FNa administration timing and dosing as structured as possible. In this study, we have not investigated the effect of the FNa redosing on wide-field operating microscope imaging results or its effectiveness in optimizing surgical resection.

Although most artifacts were excluded in the image analysis, red blood cells can still produce dark signals that may appear on CLE images with a different frequency. An abundance of such artifacts can directly affect the mean brightness and contrast of the image. In addition, alterations of the image acquisition filter systems should be widely explored to alter and improve image characteristics. As experience with CLE increases, more sophisticated technology for image processing and implementation of machine learning for image interpretation may be used.

## 5 Conclusions

The decay of the FNa signal revealed by CLE *ex vivo* imaging was directly associated with image intensity. Image brightness, contrast, and quality, and therefore interpretation, were improved with FNa redosing. However, given the diversity of factors influencing FNa metabolism and the individual surgical situation, the decision to redose a patient with FNa should be made for every clinical situation uniquely. This study demonstrates that FNa redosing, at least at 2 to 5 mg/kg, should be considered a safe and beneficial manipulation during prolonged resections of tumors and where CLE imaging is employed to interrogate the tumor and associated tissue region.

## Data Availability Statement

The raw data supporting the conclusions of this article will be made available by the authors, without undue reservation.

## Ethics Statement

The studies involving human participants were reviewed and approved by the Institutional Review Board for Human Research at St. Joseph’s Hospital and Medical Center. The patients/participants provided their written informed consent to participate in this study.

## Author Contributions

Study planning and coordination: MCP, IA. Acquisition of confocal images: EB. Processing and organizing of the data and confocal images: IA, AD, and MCP. Assessment of confocal images: IA, AD, MTP, LB, and MCP. Statistical analysis: IA. Writing a draft: IA, MTP, AD, and MCP. Review of the draft: all authors. Final approval: MCP. All authors contributed to the article and approved the submitted version.

## Funding

This study was supported by funds from the Newsome Chair in Neurosurgery Research held by MCP and from Barrow Neurological Foundation.

## Conflict of Interest

The authors declare that the research was conducted in the absence of any commercial or financial relationships that could be construed as a potential conflict of interest.

## Publisher’s Note

All claims expressed in this article are solely those of the authors and do not necessarily represent those of their affiliated organizations, or those of the publisher, the editors and the reviewers. Any product that may be evaluated in this article, or claim that may be made by its manufacturer, is not guaranteed or endorsed by the publisher.

## Abbreviations

5-ALA, 5-aminolevulinic acid; BBB, blood–brain barrier; CLE, confocal laser endomicroscopy; FDA, Food and Drug Administration; FNa, fluorescein sodium; H&E, hematoxylin and eosin; ICG, indocyanine green; NIR, near infrared; PpIX, protoporphyrin IX; SD, standard deviation.
